# Supplementary material for: In Situ Synthesis of NiFeLDH/A–CBp from Pyrolytic Carbon as High-Performance Oxygen Evolution Reaction Catalyst for Water Splitting and Zinc Hydrometallurgy
Source: Materials (Basel). 2023 May 26;16(11):3997. doi: 10.3390/ma16113997 (PMC10254529; doi:10.3390/ma16113997)
Supplement: Supplementary file 1 [file materials-16-03997-s001.zip › materials-2356267-supplementary.pdf]

In Situ Synthesis of NiFeLDH/A−CBp from Pyrolytic Carbon as High-Performance Oxygen Evolution Reaction Catalyst for Water Splitting and Zinc Hydrometallurgy

Kai Che, Man Zhao *, Yanzhi Sun and Junqing Pan *

State Key Laboratory of Chemical Resources Engineering, College of Chemistry, Beijing University of Chemical Technology, Beijing 100029, China; chekai121@163.com (K.C.); sunyz@mail.buct.edu.cn (Y.S.)
* Correspondence: 2022500073@buct.edu.cn (M.Z.); jqpan@buct.edu.cn (J.P.)

1.1 Electrochemical Measurement

The applied potential vs. Hg/HgO was converted into that vs. reversible hydrogen electrode (RHE) by the following formula (S1):

$$E_{vs.RHE} = E_{vs.Hg/HgO} + E^{\theta}_{VS.Hg/HgO} + 0.0591 \times pH \qquad (S1)$$

Electric double-layer capacitance (C_{dl}) can be calculated by the following equation (S2):

$$i_c = C_{dl}\frac{d\psi}{dt} \qquad (S2)$$

Where $\frac{d\psi}{dt}$ is the unit scan speed.

The polarization of the electrode reflects the obstruction of the electrode process, represented by the Tafel slope, as expressed in equation (S3):

$$\eta = a + b \times \log|I| \qquad (S3)$$

Where b is the Tafel slope. η is the overpotential, I is the current density, and a represents the overpotential value when the current density is measured in units (1 A·cm^{-2}).

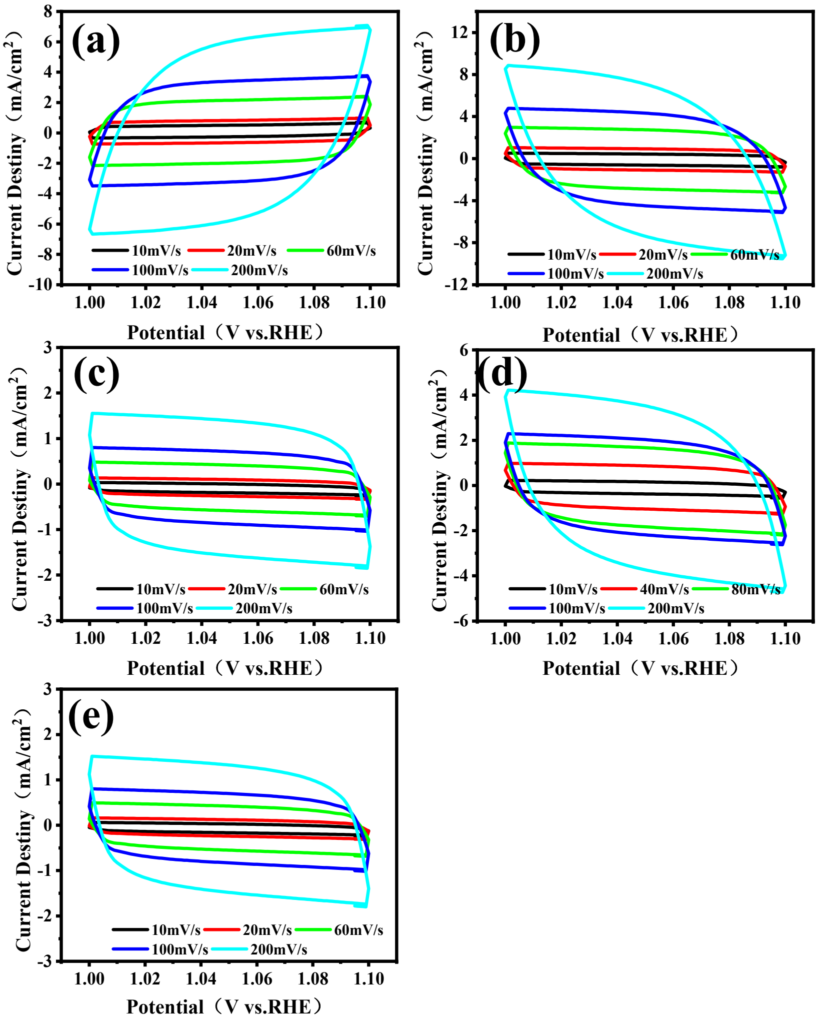

Figure S1. CV curves of NiFeLDH/A−CBp at different sweep speeds with different AA content. (a) 0 mmol, (b) 0.1 mmol, (c) 0.2 mmol, (d) 0.3 mmol, and (e) 0.4 mmol.

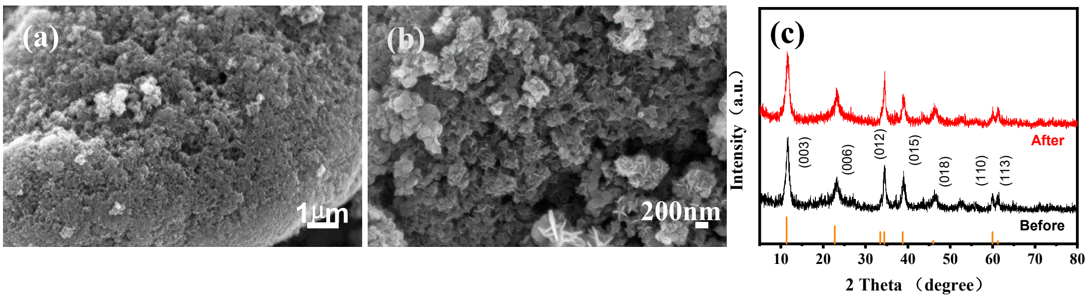

Figure S2. (a, b) SEM images of NiFeLDH/A−CBp after OER durability test. (c) XRD before and after OER durability test.

Table S1. The characterization of physical and electrochemical for as-prepared samples .

Techniques	Instrument model	Company/Country	Conditions
Scanning electron microscopy (SEM)	S-4700 model scanning electron microscope	ZEISS/Germany	Operating voltage of 20 kV
High-resolution transmission electron microscopy	JEM-2100F model high-resolution transmission electron micro-	JEOL/Japan	Operating voltage of 120 kV

(HR-TEM)	scope		
X-ray diffraction (XRD)	Ultima IV	Rigaku Industrial Corporation/Japan	2θ ranging from 5-90° with Cu Kα radiation at scan of 10° min^{-1}
X-ray photoelectron spectroscopy (XPS)	ESCALAB 250	Thermo Fisher Scientific/USA	Al Kα radiation
Fourier Transform infrared spectroscopy (FTIR)	Thermo Scientific Nicolet iS10	Nicolet /USA	KBr tabletting
Cyclic voltammetry (CV)	CHI 760D	Shanghai Chenhua Instrument, China	Potential window from 1 to 1.1 V vs. Hg/HgO
Electrochemical impedance spectroscopy (EIS)	CHI 760D	Shanghai Chenhua Instrument, China	Frequency range from 0.01 Hz to 100,000 Hz with 5 mV amplitude.
Linear sweep voltammetry (LSV)	CHI 760D	Shanghai Chenhua Instrument, China	the potential range of 1.1−1.8 V (vs. RHE) at the scan rate of 5 mV • s^{-1}

Table S2. Comparison of catalysts with high OER activity in alkaline solution reported in various literature.

	Overpotential（mV）
This work	227
CoMoV LDH [16]	270
F-Co_3Fe LDH[17]	276
Fe-NiNC-50[18]	340
$Ni_{1.5}Co_{0.5}$@NC NT/NFs[19]	243
GDY@NiFe[20]	260
